# Efficient Removal of Co(II) Ions from Aqueous Solutions Using Polyampholyte Resin: Synthesis, Properties, and Performance

**DOI:** 10.1021/acsomega.4c09127

**Published:** 2025-01-23

**Authors:** Justyna Ulatowska, Łukasz Stala, Agnieszka Kowalska, Sylwia Haor, Izabela Polowczyk

**Affiliations:** Department of Process Engineering and Technology of Polymers and Carbon Materials, Wroclaw University of Science and Technology, 27 Wybrzeże Wyspiańskiego Street, Wrocław 50-370, Poland

## Abstract

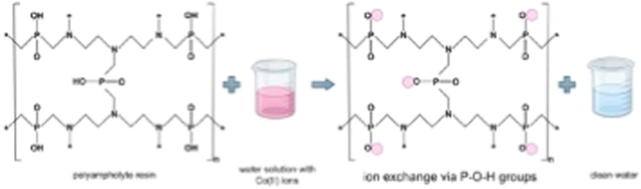

This paper presents the properties of a novel polyampholyte resin synthesized through the phosphinomethylation of diethylenetriamine. The resin, derived from inexpensive and safe building blocks, avoids the typical crude-oil resin matrix, such as poly(DVB), offering a notable advantage over commercially available solutions. Moreover, the synthesis process is straightforward and environmentally benign, aligning with the principles of sustainability and environmental protection. The primary objective of this study was to evaluate the efficiency of Co(II) ion removal from aqueous solutions using the synthesized resin under both static and kinetic conditions. Key parameters, including the initial metal ion concentration, pH, temperature, contact time, and resin dosage, were systematically investigated. A comprehensive mathematical analysis of static, kinetic, and thermodynamic parameters confirmed the effectiveness of the polyampholyte resin in removing Co(II) ions from aqueous solutions. Data analysis using the Langmuir isotherm model revealed a maximum sorption capacity of 191.7 mg/g at 328 K. Kinetic data were assessed using pseudo-first order, pseudo-second order, and Elovich kinetic models, while the Weber-Morris model was employed to determine the rate-controlling step in the Co(II) ion removal process. The results indicated that the removal of Co(II) ions follows a pseudo-second-order kinetic model, suggesting chemisorption as the dominant mechanism, with diffusion identified as the rate-controlling step according to the Weber-Morris model. Thermodynamic analysis demonstrated that the removal of Co(II) ions is spontaneous and endothermic (Δ*H* = 24.83 kJ/mol), with efficiency increasing at higher temperatures. Desorption studies using various reagents showed that 2 M H_2_SO_4_ achieved the maximum desorption of Co(II) ions (98%). The high ion removal efficiency and ease of regeneration make the synthesized resin a competitive alternative to currently available commercially adsorbents. Notably, the use of this novel polyampholyte resin represents a significant advancement in environmental protection (through reduced reliance on crude oil derivatives) and water treatment (via the removal of toxic ions).

## Introduction

Environmental pollution by heavy metals represents a significant global concern due to its widespread and severe impact. The term heavy metals encompasses elements utilized in various industries, concurrently posing threats to human health and the environment due to their inherent toxicity. These metals depending on classification include metals (e.g., cadmium, chromium, cobalt, copper, lead, mercury, nickel, zinc), semimetals (e.g., arsenic, tellurium), and even nonmetals (selenium).^1,2^ Their nonbiodegradable nature leads to persistent accumulation in the environment, posing a substantial risk to human life through the food chain.^3^

Cobalt, among these heavy metals, is particularly noteworthy for its extensive use in nuclear and coal-fired power plants, as well as in diverse industries like mining, metallurgy, electroplating, petrochemicals, battery and semiconductor manufacturing, paint, and pigment production, and in the pharmaceutical and food industries.^4−6^ Despite its beneficial role as a micronutrient and a component of cobalamin (Vit. B12), which regulates erythrocyte production, excessive intake of cobalt can lead to toxicity and carcinogenic effects.^5^ Elevated levels of cobalt in the body may result in various health issues, including paralysis, diarrhea, asthma, pneumonia, weight loss, vomiting and nausea, and damage to the thyroid and liver.^7,8^

To address the environmental challenge posed by heavy metals like cobalt, various methods for their removal from aqueous solutions have been developed. These methods encompass precipitation, adsorption, ion exchange, membrane separation and electrochemical techniques.^1,9,10^ Sorption processes, particularly adsorption and ion exchange, play a dominant role in water and wastewater treatment.^11^ Ion exchange, occurring on ionites or water–insoluble solids, involves the reversible exchange of ions in solution (e.g., heavy metal ions) for ions of the same sign, which are attached to the ionite.^12^ The efficiency of ion exchange depends on several factors, including the chemical properties of the resin, the type and concentration of ions removed, the pH of the solution, and the duration of the process. Despite its advantages, such as minimal waste generation, easy resin regeneration, and metal recovery, ion exchange is hindered by its high cost.^13^ The materials used in ion exchange can be categorized as organic and inorganic, and natural or synthetic.^1,8,14^ Synthetic ion exchange resins, particularly copolymers of polystyrene and divinylbenzene in cationites and anionites forms are widely employed.^15−17^ Another less common but promising group of ion-exchange resins is polyampholytes, which, besides their proven sorptive properties, exhibit buffering capabilities.^18−20^ A range of commercial polyampholyte resins is used to remove toxic metal ions and can be classified into two main groups. The first group consists of resins containing iminodiacetic groups, such as Chelex 100, Purolite S930, Amberlite IRC-718, and Levatite TP-207.^16,21−24^ The second group includes resins with aminomethylphosphonium groups, including Purolite 930, 940 and 950, Amberlite IRC-747, and Levatite TP-260.^16,25−28^

This paper focuses on the synthesis, characterization, and application of polyampholyte resin for the removal of Co(II) ions from aqueous solutions. The polyampholyte resin is synthesized through the phosphinomethylation of diethylenetriamine, incorporating hypophosphite groups that bind metal cations and nitrogen heteroatoms that bind anions. The reactions involved are straightforward and environmentally benign, aligning with environmental protection and sustainability principles. The described material, derived from inexpensive and safe building blocks, does not use the typical ion-scavenger matrix from crude oil intermediates like poly(DVB), offering an advantage over commercially available solutions.

The primary aim of the research was to develop an efficient and greener material for removing toxic metal ions. The resulting product, the polyampholyte resin, was characterized by its functional groups (using Fourier transform infrared (FTIR)), surface chemical composition (using X-ray photoelectron spectroscopy (XPS)), and physicochemical properties (such as swelling, density, and specific surface area). A series of experiments were conducted to evaluate the efficiency of the polyampholyte resin in removing Co(II) ions from aqueous solutions under both kinetic and static conditions. Various parameters, including initial metal ion concentration, pH, temperature, and resin dosage, were investigated to optimize the removal process. Mathematical modeling of the process’s static, kinetic, and thermodynamic parameters led to the proposal of a mechanism for binding Co(II) ions to the polyampholyte resin. Detailed characterization and application tests enabled the determination of the sorption efficiency of the new resin, which could serve as a competitive alternative to currently used ion exchange resins, primarily due to the reliance on crude oil-derived materials.

## Materials and Methods

### Materials

Cobalt(II) chloride hexahydrate (ACS reagent, ≥98%, CAS No. 7791-13-1) and nitroso-R salt (pure, CAS No. 525-05-3) were obtained from Sigma-Aldrich. Diethylenetriamine (96%, CAS No. 4097-89-6) and paraformaldehyde (extra pure, 96%, CAS No. 30525-89-4), purchased from Acros Organics, along with hypophosphorous acid (50%, CAS No. 6303-21-5) from Sigma-Aldrich, were utilized in the synthesis of the polyampholyte resin. Other reagents, employed for preparation and analysis, were procured from Avator Performance Materials Inc., and used without any pretreatment.

### Analyses Methods

#### NMR

The spectra of ^1^H and ^31^P NMR (400 and 162 MHz, respectively) were collected on a Jeol 400yh NMR spectrometer (JEOL USA, Inc., Peabody, Massachusetts).

#### SSA

The specific surface area of the examined polyampholyte resin was determined through single-point analysis utilizing a Flow Sorb 2300 apparatus (Micromeritics Instruments Corp, Norcross, Georgia) and involved low-temperature nitrogen sorption from a mixture containing 30% nitrogen and 70% helium, followed by subsequent desorption.

#### Images

Three types of images were captured using (i) a digital camera; (ii) an Axio Imager.M1m optical microscope (Zeiss, Jena, Germany); (iii) and a JSM-6610LV scanning electron microscope (JEOL Ltd., Akishima, Japan) after sputtering the samples with carbon using a JEC-530 automatic coating machine (JEOL Ltd., Akishima, Japan).

#### Electrokinetic Potential

The ζ-potential was determined using a Zetasizer 2000 apparatus (Malvern Instruments Ltd., Malvern, UK). Electrokinetic potential measurements were conducted within the pH range of 1.50–11.50. Solution pH was controlled by adding either 0.1 or 1 M hydrochloric acid solution for acidic conditions or 0.1 or 1 M sodium hydroxide solution for basic conditions. The reported electrokinetic potential values represent the averages of five measurements.

#### FTIR

The FTIR spectra in KBr pellets were recorded using a VERTEX 70v spectrometer (Bruker, Billerica, Massachusetts) within a wavenumber range of 400–4000 cm^–1^.

#### XPS

The XPS analyses were performed using a PHI 5000 VersaProbeII XPS/UPS photoelectron spectrometer (ULVAC-PHI, Chigasaki, Japan) with monochromatic Al Kα (1486.6 eV) X-rays focused on a 100 μm spot. The scanning area was 400 μm × 400 μm. The photoelectron takeoff angle was set to 45°, with the pass energy of 117.50 eV for survey scans and 46.95 eV for high-energy resolution spectra of the C 1s, O 1s, N 1s, and P 2p regions. The operating pressure in the analytical chamber was maintained at less than 2 × 10^–9^ mbar.

#### Cobalt Concentration

The concentrations of Co(II) ions in the solutions remaining after adsorption were determined using the UV–visible spectrophotometer Evolution 201 (Thermo Fisher Scientific, Madison, USA) employing the nitroso-R salt method, following the standard procedure at analytical wavelengths of 415 nm.^29,30^ The sorption Co(II) capacity of polyampholyte material was calculated using the eq 1
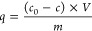
1The percentage removal of Co(II) was calculated using eq 2
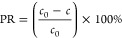
2where *c*_0_ is the initial concentration of Co(II) (mg/dm^3^), *c* is the concentration of Co(II) (mg/dm^3^), *m* is the polyampholyte mass (g) and *V* the solution volume (dm^3^).

### Synthesis of Polyampholyte Resin

**Figure 1:**
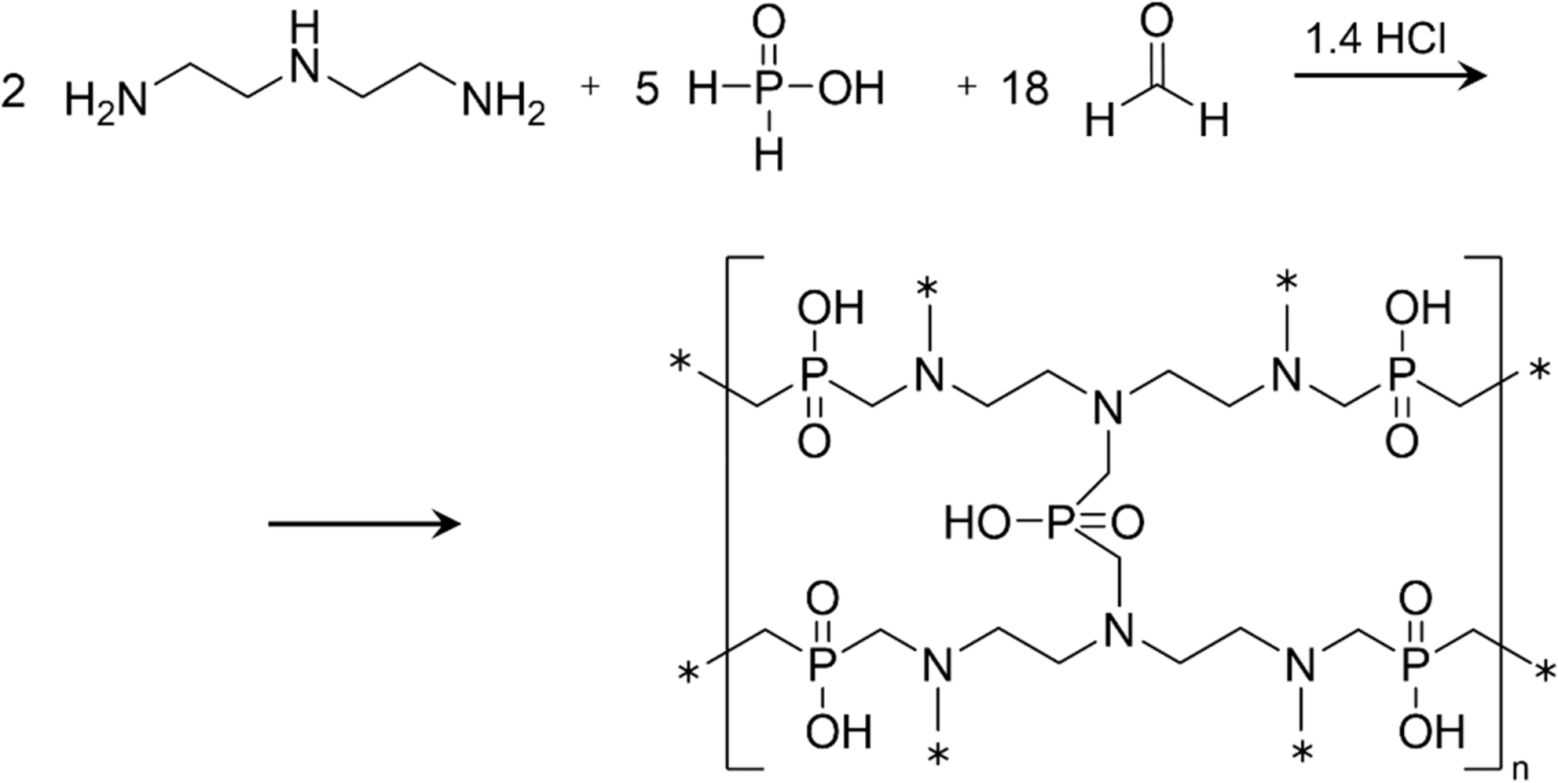
Synthesis of polyampholyte resin.

10.329 g of diethylenetriamine and 36 cm^3^ of distilled water were placed into a round-bottom flask. The flask was set on a tripod above a magnetic stirrer with a heating plate. A reflux condenser and thermometer were attached to the flask. With stirring initiated at 500 rpm, a total of 15 cm^3^ of concentrated hydrochloric acid was added in three portions. Next, 26 cm^3^ of 50% phosphinic acid, 27.019 g of paraformaldehyde, and 60 cm^3^ of distilled water were added to the mixture. The heating was then started at 373 K. The reaction scheme is shown in Figure 1.

After 60 min, a sample was taken from the solution above the resin for ^31^P and ^1^H NMR analysis. The resulting resin was crushed and washed in several portions with approximately 50 cm^3^ of distilled water every 30 min. After removing residual unreacted substrates and adjusting the filtrate’s pH to around 7, the resin was dried at 373 K. The dry resin was ground and sieved through a sieve (*d* = 0.5 mm).

### Utilization of Polyampholyte Resins for Cobalt Removal from Aqueous Solutions

#### Effect of pH on Co(II) Ions Removal

A 20 mg resin was weighed into each of the eight test tubes. Subsequently, 10 cm^3^ of 590 mg/dm^3^ cobalt stock solution with varying pH values ranging from 1.0 to 7.5 was added to each tube. The pH of the solution was adjusted by adding 1 M hydrochloric acid solution or 1 M sodium hydroxide solution. The prepared samples were then placed in a laboratory incubator equipped with shaking (150 rpm). After 24 h, the extent of Co(II) ion removal from the solution by the resin was analyzed. Additionally, the pH of the solution in the samples was checked after 24 h.

#### Effect of Resin Dose on Co(II) Ions Removal

An assigned quantity of resin (varying from 7.5 to 100 mg) was carefully weighed and placed into 11 test tubes. Subsequently, 10 cm^3^ of a 590 mg/dm^3^ cobalt stock solution was added to each tube. The prepared samples were placed in a laboratory incubator equipped with shaking at 150 rpm. After 24 h, the level of removal of Co(II) ions from the solution over the resin was analyzed.

#### Effect of Initial Concentration and Temperature on Co(II) Ions Removal

A 20 mg resin was weighed into ten test tubes. Subsequently, 10 cm^3^ of each cobalt solution, with a specified initial concentration ranging from 120 to 1180 mg/dm^3^, was introduced into the tubes. The prepared samples were then placed in a laboratory incubator equipped with shaking (150 rpm), with the temperature set to either 298, 308, 318, or 328 K. After 24 h, the concentration of cobalt ions in the samples was analyzed.

The isothermal data obtained were subsequently fitted to the Langmuir mathematical model using the linear form of this eq 3(31,32)
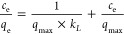
3where *c*_e_ is the equilibrium concentration of Co(II) ions (mg/dm^3^); *q*_e_ is the equilibrium sorption capacity of the resin (mg/g); *q*_max_ is the maximum capacity of the monolayer (mg/g), and *k*_L_ is the equilibrium sorption constant related to the characteristic energy of the energy distribution function.

To calculate the enthalpy change (Δ*H*), entropy change (Δ*S*), and change Gibbs free energy (Δ*G*^o^) were used from the Van’t Hoff eq 4 and known relationship (eq 5)^33^
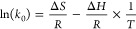
4
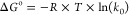
5where *k*_0_ is the pre-exponential factor related to the entropy of the process, *R* is the universal gas constant (8.314 J/(mol K)), and *T* is the temperature (K). The thermodynamic equilibrium constant *k*_0_ for the process was obtained by plotting ln(*c*/*c*_equiv_) versus *c*_equiv_ (where *c* is the equilibrium Co(II) concentration on the polyampholyte material (mg/dm^3^) and is calculated as *c*_0_*–c*_equiv_) and extrapolating to zero using a graphical method. From the intersections of plots with the vertical axis, the value of ln(*k*_0_) was estimated at the four different temperatures (298, 308, 318, and 328 K).

To assess the suitability of the resin for the sorption of Co(II) ions, a metric known as the separation coefficient was employed, defined by the following eq 6(34,35)
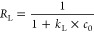
6where the *k*_L_ is the adsorption equilibrium constant determined from the Langmuir equation, and *c*_0_ is the initial concentration of Co(II) ions in solution (mg/dm^3^). The parameter *R*_L_ indicates the shape of the isotherm accordingly: *R*_L_ > 1, unfavorable; *R*_L_ = 1, linear; 0 < *R*_L_ < 1, favorable; *R*_L_ = 0, irreversible.^34,36^

#### Effect of Contact Time on Co(II) Ions Removal

1000 mg of resin was weighed and placed into a beaker, and then 100 cm^3^ of a 590 mg/dm^3^ cobalt stock solution was poured in. The prepared suspension was placed on a magnetic stirrer (150 rpm), and measurements began. At specified intervals, samples were taken for Co(II) ion concentration analysis, starting at 1 min and ending after 24 h. The level of ion removal in the samples was analyzed.

Four kinetic models were employed to examine the kinetic of Co(II) ions sorption: the pseudo-first-order model (eq 7), the pseudo-second-order model (eq 8), the Elovich model (eq 9), and the Weber–Morris model (eq 10)^37−39^
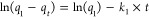
7
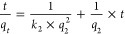
8
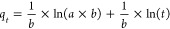
9
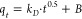
10where *q*_t_ is sorption capacity in any time (mg/g), *k*_1_ is the rate constant of the pseudo-first-order kinetic model (1/min), *k*_2_ is the rate constant of the pseudo-second-order kinetic model (g/(mg min)), a is the maximum sorption capacity (mg/g), *b* is Elovich equation constant (dm^3^/mg), *k*_D_ is intraparticle diffusion rate constant (mg/(g min^0.5^)), and *B* is boundary layer thickness (mg/g).

The determination coefficient (*R*^2^), the most common measure used in classical linear regression, was employed to measure the model’s fit to the experimental data. The determination coefficient was estimated based on the relationship (eq 11)
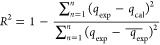
11where *q*_exp_ is the sorption capacity of the resin obtained experimentally (mg/g), and *q*_cal_ is the sorption capacity of the resin obtained from the model (mg/g).

#### Regeneration of Resin

The regeneration study utilized a resin that had previously adsorbed Co(II) ions. It was obtained by mixing 5 g of polyampholyte resin with 100 cm^3^ of a 1180 mg/dm^3^ cobalt solution. The resin and the solution were allowed to come into contact for 24 h through stirring at 150 rpm. After this period, the solid was separated from the solution using a filter and washed several times with demineralized water. The resin with Co(II) ions was subsequently dried and utilized for later experiments. For the regeneration studies, six regeneration agents were employed: 6 and 1 M hydrochloric acid solution, 2 and 1 M sulfuric acid solution, 1 M sodium hydroxide solution, and demineralized water. In six test tubes, 0.5 g of resin was weighed after Co(II) adsorption, poured with demineralized water (2 cm^3^), and allowed to swell for several minutes. The samples were then treated with the appropriate regeneration agent. Prepared samples were placed in a laboratory incubator with shaking (150 rpm). After 24 h, the level of removal of Co(II) ions from the solution over the resin was analyzed.

## Results and Discussion

### Characteristics of the Resin

The conducted synthesis resulted in a polyampholyte resin with the structural formula depicted in Figure 1, comprising 7.6 mol/kg of –P(O)OH groups and 9.1 mol/kg of –N(CH_2–_*)_3_ groups. The molar mass of the resin mer (C_18_H_41_N_6_O_10_P_5_)*_n_* is 656,4 g/mol.

Analysis of the obtained ^31^P NMR spectrum of the solution above the resin indicated that the presence of phosphoric acid (0.4s), phosphinic acid (5.1d), and soluble polymeric compounds (8.5, 11.9, 15.4, 16.2). Similarly, the analysis of the ^1^H NMR spectrum of the solution reveals peaks from soluble hydroxyphosphonic polymers (4.6). ^31^P NMR and ^1^H NMR spectra were included in Supporting Information (Figures S1 and S2, respectively).

**Figure 2:**
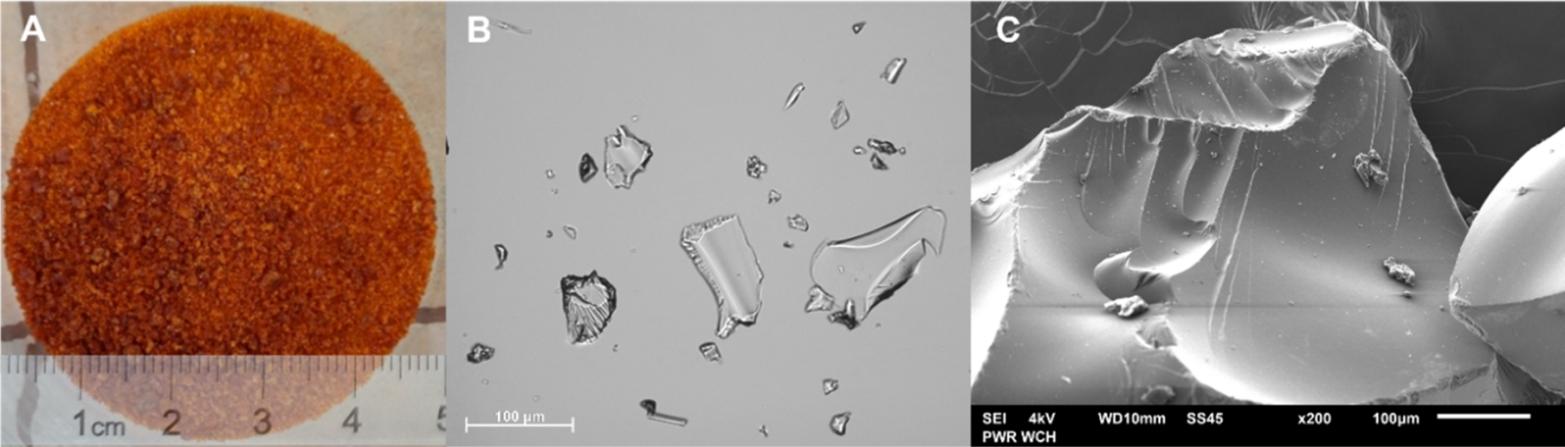
Images of the obtained polyampholyte resin taken with a digital camera (A), an optical microscope (B), and a scanning electron microscope (C).

The synthesis yielded a polyampholyte resin in the form of a gel, which, upon drying, resulted in an orange solid with an irregular particle shape (Figure 2). It is an ampholytic polycondensate cross-linked with methyl groups, whose functional groups are hypophosphite groups. The reaction yield for the resin was 98.8% on a dry-weight basis. The obtained resin has a small specific surface area of about 0.2 m^2^/g and a 2550 g/dm^3^ density.

Swelling under a solvent is a characteristic property of various natural and synthetic materials. Polymeric ion exchange resins contain numerous hydrophilic groups and exhibit swelling upon contact with water. The extent of swelling is influenced by the ionite’s structure, the strength of bonds in its composition, and the physical and chemical characteristics of the solution it comes into contact with.^40−42^ Swelling tests were conducted to observe the volume increase of the resulting polyampholyte resin in water and to determine the time required for complete swelling. The results, illustrating the change in bed height over time, are presented in Supporting Information as Figure S3. Notably, after 10 min, no further change in bed height was observed. At this point, the height of the resin bed had increased to 3.2 times the initial height (*h*_0_ = 5 cm). The obtained results suggest that the easy swelling of the polyampholyte resin in water is due to the presence of hydrophilic functional groups (e.g., −OH, −COOH, −CONH_2_) in the structure of the synthesized material. Additionally, the polymer content of the swollen material was calculated as the ratio of the dry weight of resin to the wet weight, resulting in 23.6%.

**Figure 3:**
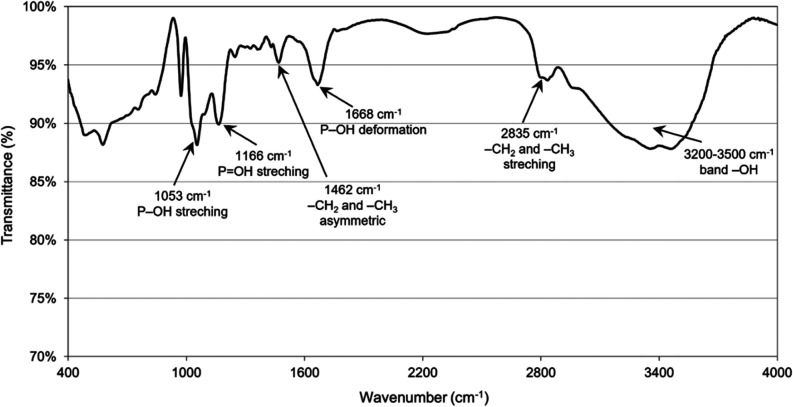
FTIR spectrum featuring characteristic peaks of the obtained polyampholyte resin.

In the FTIR spectrum of the obtained polyampholyte resin (Figure 3), a band in the hydrogen bonding range (3200–3500 cm^–1^), vibrations of C–H groups (1462 and 2835 cm^–1^), and vibrations associated with the occurrence of phosphorus groups (1053, 1166, and 1668 cm^–1^) are observed.^43^

Surface concentrations of chemical bonds determined from fitting XPS data for the analyzed polyampholyte material are listed in Table 1.

**Table 1:** Surface Composition (Atomic %) was Determined by Fitting XPS Spectra for the Analyzed Sample

element	binding energy [eV]	groups/oxidation state	surface composition [%]
C	284.3	C=C	22.7
		C–C	
	285.0	C–C	30.1
		C=N	
		C–O	
	287.3	C=O	1.2
		O–C–O	
O	529.9	O=C–N	12.5
		O=C	
	531.5	O–C	13.3
		–OH	
N	398.6	imine	5.7
		N=N=N	
	401.0	amine	5.9
		N–C=O	
		NH_4_^+^	
P	131.7	P–(−CH−)_3_	8.6

**Figure 4:**
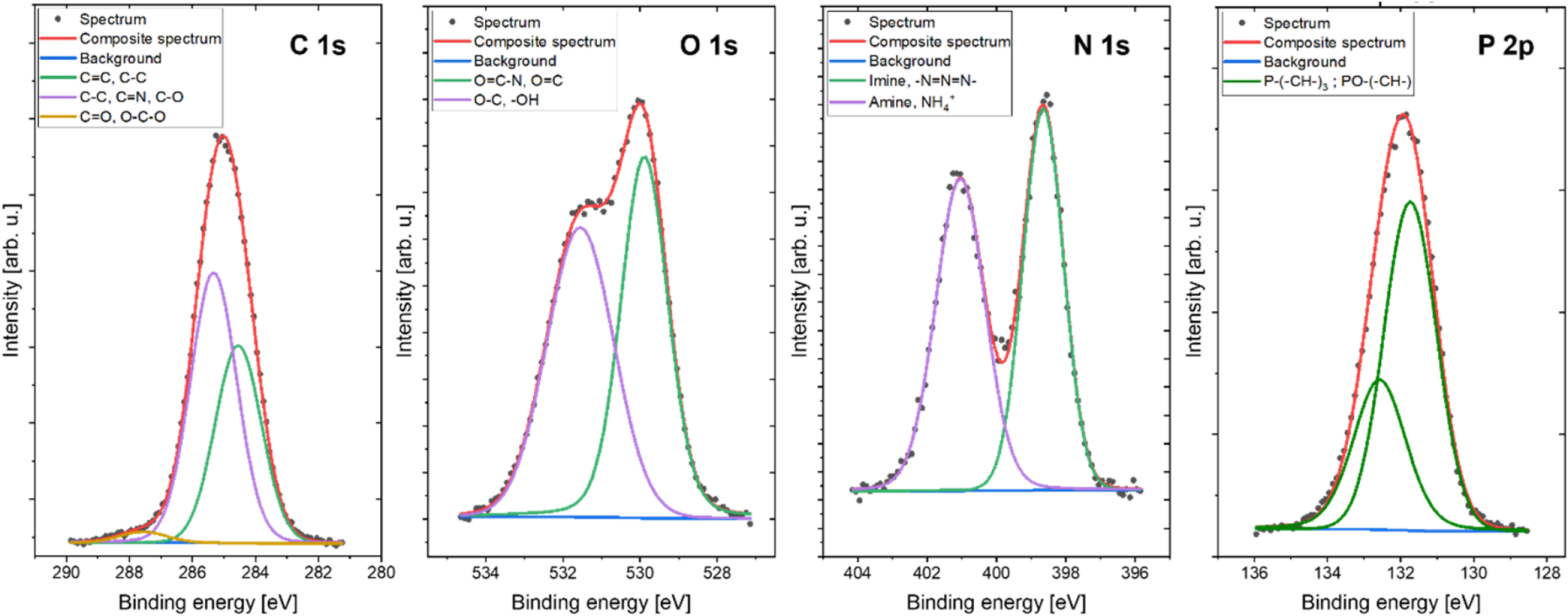
XPS spectra of C 1s, O 1s, N 1s, and P 2p photoelectrons of the obtained polyampholyte resin.

In Supporting Information, Figure S4 provides an overview of the XPS spectrum, which was used to select high-resolution scanning areas and record spectra in specific regions of photoelectron emission. The comprehensive analysis revealed the predominant presence of carbon, oxygen, nitrogen, and phosphorus. This thorough investigation led to a detailed analysis of these four elements. The XPS photoelectron spectra for C 1s, O 1s, N 1s, and P 2p are shown in Figure 4, further validating the depth of our research.

The C 1s spectra were fitted with three lines: the first one at 284.3 eV comes from C=C sp^2^ type bonds and/or some part of aliphatic carbon C–C/C–H type bonds; the second centered at 285.0 eV originates from organic C–C type bonds and/or C–O and/or C=N type bonds; and the last line positioned at 287.3 eV mainly comes from C=O and/or O–C–O type bonds.^44,45^ The O 1s spectra were fitted with two components: the first line centered at 529.9 eV points out the existence of oxygen ii organic type N–C=O and/or O=C bonds, whereas the second line centered at 531.5 eV indicates the presence of either O–C and/or – OH type compounds.^45^ The N 1s spectrum was fitted with two lines: the first centered at 398.6 eV, which indicates the presence of C–N=C type bonds in aromatic structures (imines) and/or azide type compounds, and the second line at 401.0 eV, which may originate from central three-coordinated nitrogen N–C_3_ and/or N–C=O type groups and/or NH_4_^+^ ions presence.^44,46^ The P 2p spectra were fitted with a doublet structure (2p_3/2_–2p_1/2_ splitting value is 0.8), in which the main line centered at 131.7 eV indicates the presence of organophosphines or phosphine oxide-type compounds.^46^

### Utilization of Polyampholyte Resins for Cobalt Removal from Aqueous Solutions

Sorption experiments were conducted to determine the optimal conditions for removing Co(II) ions from aqueous solutions on polyampholyte resin. Using similar process conditions, i.e., temperature (298 K), initial Co(II) concentration (590 mg/dm^3^), resin dosage (20 mg), and pH (about 7.0), the effects of individual parameters on the process and Co(II) ion removal efficiency were studied. All experiments were conducted for 24 h to ensure process equilibrium.

#### Effect of pH on Co(II) Ions Removal

The initial pH of the solution is one of the critical parameters that significantly affect the extent of Co(II) ion removal by polyampholyte resins. This influence is crucial because pH directly impacts the speciation of ions in aqueous solutions. It is noteworthy that ion speciation plays a pivotal role, especially in the case of transition metal ions like cobalt, as they can form various complexes with different ligands present in the solution. Different forms of Co(II) ions speciation can be identified depending on the pH of the solution: (i) Co^2+^ ions predominant at low pH (acidic), Co(II) ions exist mainly as Co^2+^; (ii) hydroxo complexes—as the pH increases (from acidic to neutral), Co(II) ions can form complexes with hydroxyl (OH^–^) ions derived from water dissociation, such as Co(OH)^+^; and (iii) hydroxides and hydroxide complexes—at high pH (alkaline conditions), Co(II) ions can form hydroxides, such as Co(OH)_2_, as well as hydroxide complexes, such as Co(OH)_3_^–^.^47−49^

**Figure 5:**
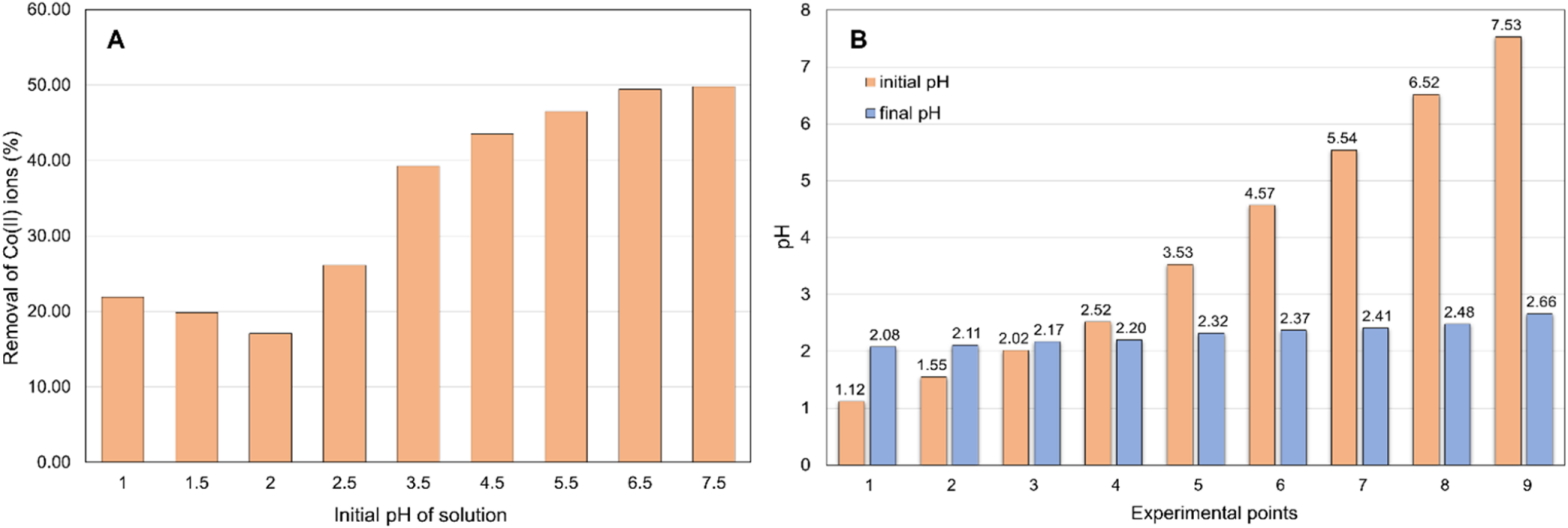
Effect of initial pH on the removal of Co(II) ions (A) and comparison of the solution pH change before and after ion exchange of Co(II) ions (B) (resin dose: 20 mg, initial Co(II) concentration: 590 mg/dm^3^, *T* = 298 K, time: 24 h).

The current study explores the impact of pH in the strongly acidic to neutral range to exclude potential Co(II) ion removal through precipitation at alkaline pH. The obtained results are depicted in Figure 5A. Results showed that with the increase of pH from 1 to 7.5, the percentage of Co(II) ion removal by the polyampholyte resin increased from 21.9 to 49.8%, respectively. At very low pH values, electrostatic interactions may impede the sorption of Co(II) ions due to both Co(II) ions and the resin surface being positively charged, which explains the limited sorption observed at low pH values. As the pH increases, causing the surface charge to become more negative, the interactions between Co(II) ions and the resin decrease, leading to enhanced Co(II) sorption. A pH of about 7.0 was selected as the optimum pH for the removal process of the Co(II) ion on polyampholyte resin at 298 K for an initial concentration of Co(II) 590 mg/dm3 and resin dose of 20 mg. After 24 h, the pH of the solutions from which Co(II) ions were removed using polyampholyte resin was remeasured. A change in the solution pH to approximately 2.50 was observed in all tested samples. This suggests that the positive Co(II) ions present in the solutions displaced the hydrogen ions from the surface of the polyampholyte resin, leading to a reduction in pH. A comparison of the pH changes in the solutions before and after the ion exchange of Co(II) ions is illustrated in Figure 5B.

**Figure 6:**
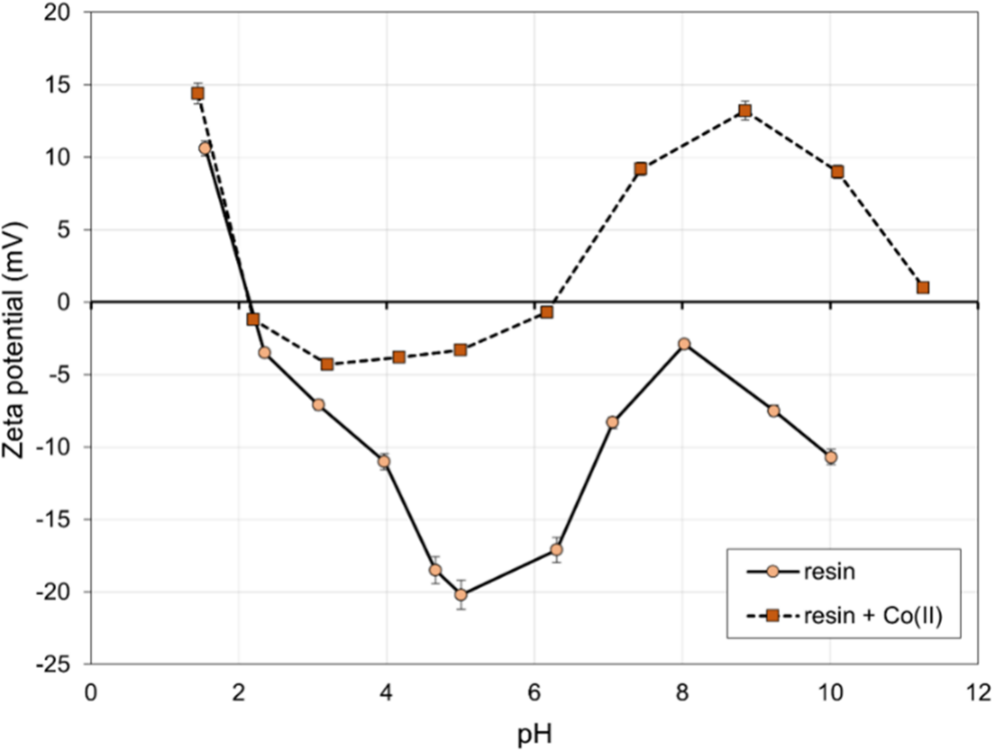
ζ-potential of the polyampholyte resin before and after contact with Co(II) ions.

The electrokinetic potential of the examined polyampholyte resin was determined both before and after the sorption of Co(II) ions, with pH being a variable parameter. Figure 6 presents the average values obtained from five replicate measurements. The standard deviations of the results varied within the range of 0.7–5 mV.

Analysis of the obtained results concludes that the electrokinetic potential remained positive at very low pH values (below 2.0). This phenomenon is likely attributable to the positive charge on the surface, preventing the attachment of positively charged Co(II) ions. With increasing pH, the potential shifts toward a negative value, thereby enhancing the sorption capacity of the material. Utilizing the resin for the removal of Co(II) ions results in a change of its negative electrokinetic potential, shifting toward zero and positive values. This change may indicate that Co(II) cations neutralized the negative resin surface charge, providing further evidence of the binding process.

#### Effect of Resin Dose on Co(II) Ions Removal

**Figure 7:**
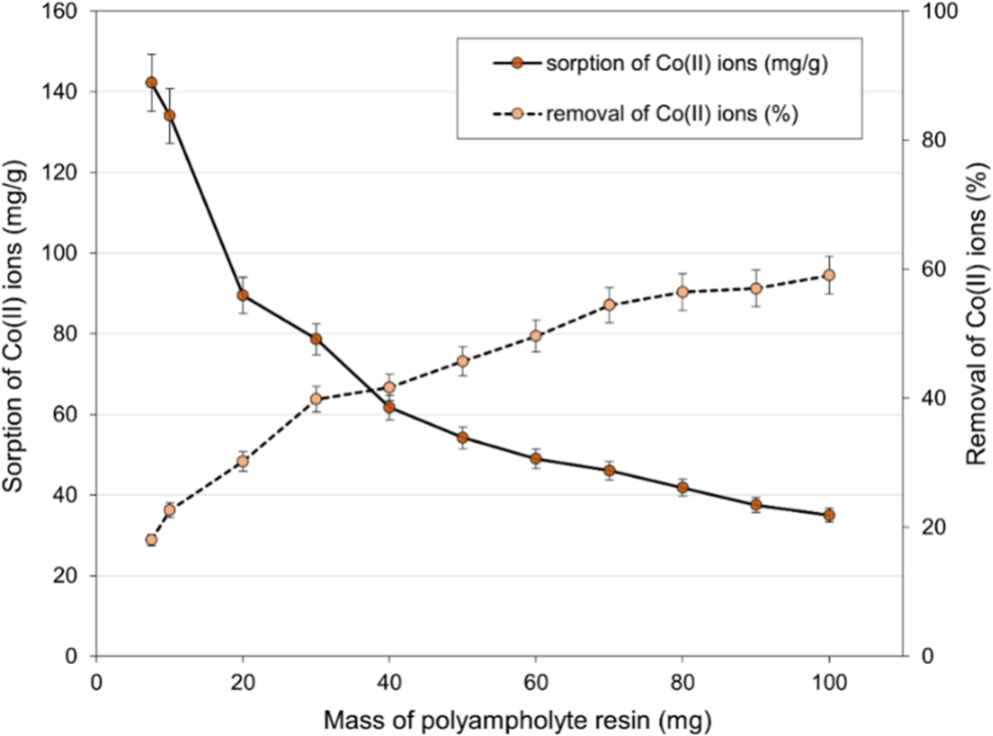
Effect of resin dose on the removal of Co(II) ions (initial Co(II) concentration: 590 mg/dm^3^, *T* = 298 K, pH ∼ 7.0, time: 24 h).

The following study on the sorption of Co(II) ions investigated how the removal rate varies with the doses of resin introduced. The results of this experiment are presented in Figure 7. The dose of resin used significantly influences both the removal rate and sorption capacity of Co(II) ions from aqueous solutions. The proper selection of the resin amount is crucial for achieving optimal process performance. An increase in the resin amount results in a higher number of available active sites, leading to an increased removal of Co(II) ions from the solution. However, it is essential to note that further increases in the resin dose may only yield marginal gains in removal levels beyond a certain point. Eventually, a point is reached where additional resin does not contribute to a higher sorption capacity. This observation is supported by the experimental data obtained.

As depicted in Figure 7, increasing the resin dose led to a proportional increase in the percentage removal of Co(II) ions from the solution. With the smallest resin amount (7.5 mg), the reduction was approximately 20%, while utilizing 100 mg resulted in a 60% removal. Further increases in the resin amount would likely lead to even greater removal of Co(II) ions from the solution, albeit at the detriment of sorption capacity. Under the tested process conditions (*c*_0_ = 590 mg/dm^3^; *T* = 298 K), the highest sorption capacity (142.2 mg/g) was achieved with the lowest resin dose. Increasing the dose led to a decline in sorption capacity, reaching 35 mg/g for a resin dose of 100 mg.

#### Effect of Initial Concentration and Temperature on Co(II) Ions Removal

**Figure 8:**
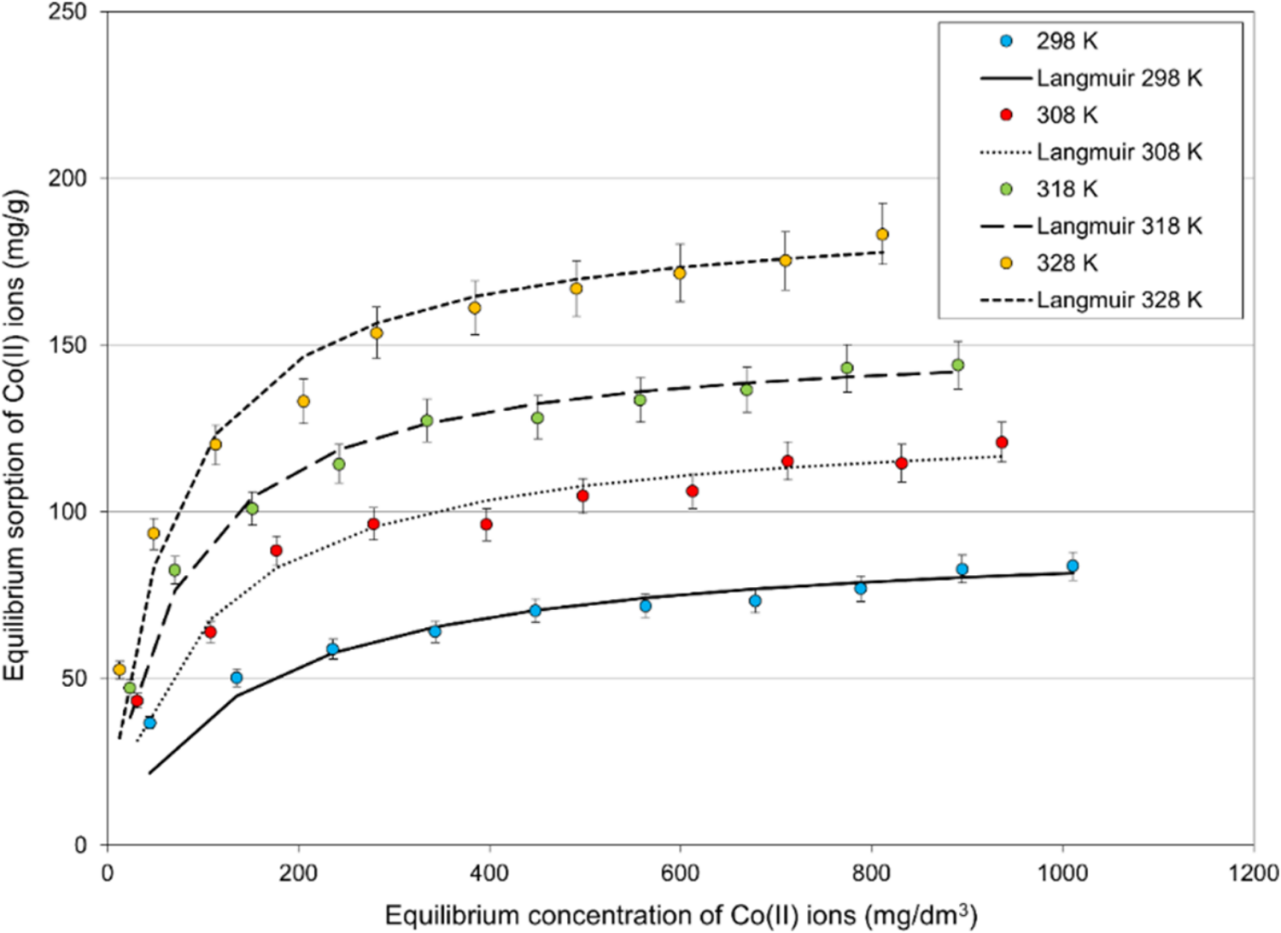
Effect of temperature on the equilibrium sorption of Co(II) ions onto polyampholyte resin (resin dose: 20 mg, *T* = 298 K, pH ∼ 7.0, time: 24 h).

Another crucial factor is the influence of the initial concentration of Co(II) ions in an aqueous solution on their removal by the polyampholyte resin. The initial concentration is crucial in reaching equilibrium and determining the maximum sorption capacity. As illustrated in Figure 8, an escalation in the initial concentration of Co(II) ions resulted in increased sorption by the studied polyampholyte resin until a certain equilibrium state was attained, indicating the maximum sorption capacity. In order to determine the effect of the initial concentration of Co(II) ions, sorption experiments were carried out to remove Co(II) ions at several initial concentrations from 120 to 1180 mg/dm^3^ at a constant resin dose (20 mg) at four temperatures at pH 7.0 and within 24 h. As a result of the experiments, the sorption capacity of Co(II) ions on the polyampholyte resin increased with the increase of the initial concentration of Co(II) ions. In these studies, the assessment of the effect of temperature on the removal rate and sorption capacity was equally important. As shown in Figure 8, increased temperature increases the sorption capacity of the polyampholyte resin. For the highest initial concentration (1180 mg/dm^3^) after 24 h, 83.7, 120.9, 143.9, and 183.3 mg/g were obtained for 298, 308, 318, and 328 K, respectively. Temperature influences the sorption equilibrium, leading to a more significant amount of Co(II) ions adsorbed by the resin at equilibrium and, consequently, to an increased rate of Co(II) ions removal from the solution at elevated temperature.

**Figure 9:**
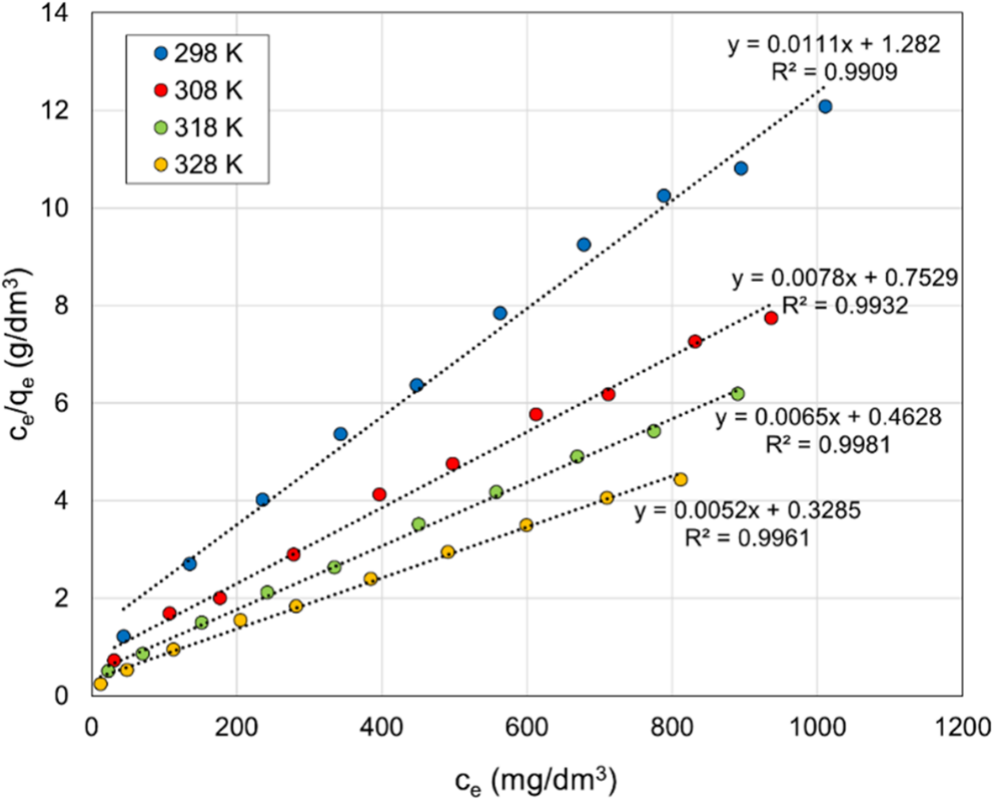
Langmuir plots for the removal of Co(II) ions.

The Langmuir isotherm model effectively represented the experimental data and approximated the experimental results very well. The high determination coefficient (*R*^2^), calculated from eq 11, indicates a strong alignment between the model and the experimental data. Figure 8 illustrates the experimental data alongside the fitting of the Langmuir model, while Figure 9 presents the linear relationships predicted by the Langmuir model.

The Langmuir model application enabled the determination of the maximum sorption capacity (*q*_max_) of the polyampholyte resin, which exhibited an increase with rising temperature. Concurrently, an observed elevation in the equilibrium constant of the process (*k*_L_) was noted with increasing temperature. The calculated parameters confirm that the removal of Co(II) ions from aqueous solutions using polyampholyte resin is more favorable at higher temperatures.

The Langmuir model parameters, along with the determination coefficient, are summarized in Table 2. A separation factor (*R*_L_) was calculated to assess the suitability of the tested resin for removing Co(II) ions from aqueous solutions. The dimensionless parameter *R*_L_ values ranging between 0 and 1, as shown in Table 2, indicate the favorable nature of the process.

**Table 2:** Calculated Langmuir Isotherm and Thermodynamic Parameters for Sorption of Co(II)

*T* [K]	*q*_*max*_ [mg/g]	*k*_L_ [dm^3^/mg]	*R*_L_ [-]	*R*^2^ [-]	ln *K*_0_	Δ**H** [kJ/mol]	Δ**S** [kJ/(mol·K)]	Δ**G** [kJ/mol]
298	93.59	0.0068	0.1111	0.993	2.415	24.83	0.104	–5.882
308	128.7	0.0130	0.0760	0.993	2.486			–6.262
318	153.3	0.0141	0.0569	0.998	3.023			–7.867
328	191.7	0.0159	0.0506	0.996	3.282			–8.814

Previous studies by the authors have demonstrated that polyampholyte resins also possess sorption capacity for other toxic metal ions. According to the Langmuir isotherm, the maximum sorption capacities of polyampholyte resins were 99.2 mg/g for Cu(II),^20^ 140.3 mg/g for Ni(II),^50^ and 459.5 mg/g for Pb(II).^51^ In comparison, the sorption capacity for Co(II) under similar process conditions (*m* = 25 mg; *T* = 298 K, pH ∼ 4.5) was found to be 93.6 mg/g. However, it is essential to note that the removal process for each of these ions becomes more favorable at higher temperatures. The experimentally determined maximum sorption capacity of the polyampholyte resin for Co(II) ions is comparable to the values reported in the literature. Table 3 provides a comparison of the sorption capacity of the synthesized resin with other resins used for Co(II) ion removal. It is worth emphasizing, however, that most resins designed for removing toxic ions from aqueous solutions are derived from petroleum-derived products.^15−17,22−26,52−57,61,62^ In light of the growing need to reduce the consumption of nonrenewable energy sources, the synthesized polyampholyte resin demonstrates significant potential for practical application.

**Table 3:** Comparison of the Sorption Capacity of the Current Polyampholyte Resin with That of Various Resins for Co(II) at 298 K

resin	sorption capacity [mg/g]	references
IRN 77 cation exchange resin	86.2	(52)
SKN 1 cation exchange resin	69.4	
chelating aminophosphonate Purolite S950	5.4–9.0	(53)
amberlite IR-120 strong acid resin	40.1	(54)
amberlite IRN-77 strong acid resin	74.6	(55)
aminophosphonic chelating resin	9.0	(26)
CR-10 chelating resin	138.5	(56)
CR-15 chelating resin	116.7	
NKC-9 strong acid resin	361.0	(57)
P(AAm-MA) resin	7.0	(58)
polymeric resin	50.8	(59)
CDAP resin	137.0	(60)
DTC resin	24.9	(61)
purolite C160	72.5	(62)
P(DADMAC-Aam)CMC/Fe_2_O_3_	70.6	(33)
P(DADMAC-SA)CMC/Fe_2_O_3_	76.2	
polyampholyte resin	93.6	this study

Conducting sorption isotherms at different temperatures allowed the determination of the thermodynamic parameters for the process. Crucial parameters for describing the removal of Co(II) ions by polyampholyte resins include the enthalpy change (Δ*H*), entropy change (Δ*S*), and Gibbs free energy change (Δ*G*^o^). These parameters were determined based on the equilibrium constant (*k*_0_) using eqs 4 and 5. The calculated thermodynamic parameters characterizing this process are summarized in Table 2. The positive enthalpy change value (Δ*H* = 24.83 kJ/mol) suggests that the removal of Co(II) ions by polyampholyte resin is an endothermic process, absorbing energy during the process. It also implies that the process becomes more favorable with increasing temperature. A positive entropy change value (Δ*S* = 0.1040 kJ/(mol K)) indicates that Co(II) ions exhibit a high degree of freedom of movement in the system, leading to an increased degree of chaos. Conversely, negative values for the change in Gibbs free energy (Δ*G*^o^) suggest that the process occurs spontaneously. The decreasing trend with increasing temperature enhances the likelihood of efficient removal of Co(II) ions by polyampholyte resin at higher temperatures.^36,63^ As reported in the literature, the removal of Co(II) from aqueous solutions using various resins is typically an endothermic process that occurs spontaneously and is more favorable at higher temperatures.^33,57,59−61^

#### Effect of Contact Time on Co(II) Ions Removal

**Figure 10:**
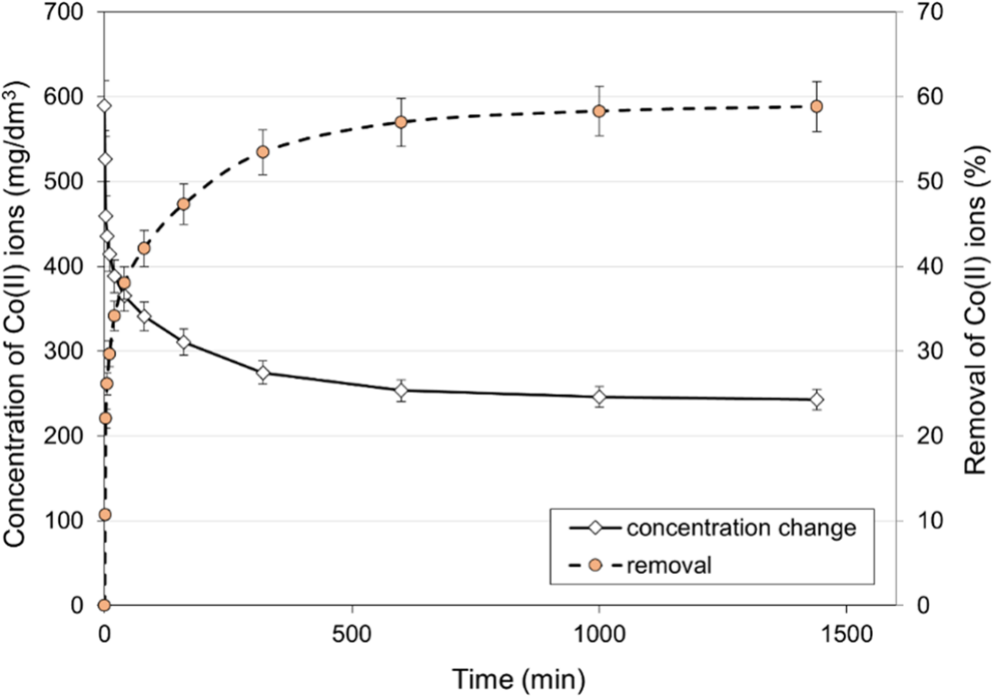
Effect of contact time on the removal of Co(II) ions (initial Co(II) concentration: 590 mg/dm^3^, *T* = 298 K, pH ∼ 7.0, time: 24 h).

The kinetics of the process is a crucial factor, prompting the conduction of kinetic studies. These experiments involved exposing polyampholyte resin to a Co(II) solution (590 mg/dm^3^, *T* = 298 K, pH 7.0) for a specified duration. Figure 10 illustrates the sorption of Co(II) ions on the examined resin. The graph indicates a reduction in Co(II) ions in the solution until equilibrium between the adsorbed Co(II) ions on the resin surface and those remaining in the solution. Once equilibrium is attained, there is no significant change in the concentration of Co(II) ions, signifying the saturation of available active sites on the resin surface. The initial stage of the process is rapid, gradually slowing down after reaching equilibrium, a typical occurrence due to available active sites saturation. The experimental results (Figure 10) show that the process of sorption of Co(II) ions on the resin is time-consuming, and equilibrium is reached only after 10 h. Although equilibrium began to be established after 10 h, it was decided that the optimal contact time would be 24 h. After this time, we are sure that equilibrium has been reached, and percentage Co(II) removal was achieved at about 58.6%. The obtained value correlates with the results obtained when testing the effect of resin dose on the removal of Co(II) ions; for a resin concentration of 10 g/dm^3^, the percentage removal of Co(II) ions from the aqueous solution was 59.0% (Figure 7).

Various kinetic models were employed to investigate the mechanism and determine the rate-controlling stage of the sorption process of Co(II) ions. The rate constants of the process were calculated utilizing the pseudo-first-order model, the pseudo-second-order model, and the Elovich model. Additionally, the Weber–Morris model (intraparticle diffusion) was used to identify the rate-controlling step. The selection of these kinetic models is justified, as they are commonly used to describe sorption kinetics and typically yield high correlation coefficients.^39^

Upon comparing experimental values with model predictions, it is evident that the pseudo-first-order model poorly describes the sorption process of Co(II) ions, as indicated by a determination coefficient (*R*^2^) of 0.975. This lesser fit is likely attributed to the rapid sorption in the initial stage of the process and the subsequent system pursuit of equilibrium.^64^ In contrast, linearization of the pseudo-second-order model yielded a highly favorable fit between experimental and model values (*R*^2^ = 0.999). The PSO model best reproduces the experimental data among the analyzed models, suggesting that chemisorption is the rate-limiting step, assuming the involvement of valence interactions or electron exchange between the adsorbate and the resin.^65^ The Elovich model also affirmed chemisorption,^66^ which exhibited a good correlation with experimental values (*R*^2^ = 0.991). Notably, the constant *a* in the Elovich model is also employed to evaluate the sorption capacity of the material under investigation.^66^ The validity employment of the three kinetic models for the experimental data followed the order: PSO > Elovich > PFO. Table 4 presents the calculated parameters of the kinetic models alongside the correlation coefficient (*R*^2^), indicating the agreement between experimental values and model predictions.

**Table 4:** Calculated Kinetic Parameters for Sorption of Co(II)

kinetic model	the pseudo-first-order model (PFO)		the pseudo-second-order model (PSO)		the Elovich model	
parameter	*q*_1_ [mg/g]	18.65	*q*_2_ [mg/g]	34.92	*a* [mg/(g min)]	31.62
	*k*_1_ [1/min]	0.00433	*k*_2_ [g/(mg min)]	0.00155	*b* [g/mg]	0.2553
	*R*^2^ [-]	0.9754	*h*_0_ [mg/(g min)]	1.894	*R*_E_ [-]	0.11
			*R*^2^ [-]	0.9992	*R*^2^ [-]	0.9905

**Figure 11:**
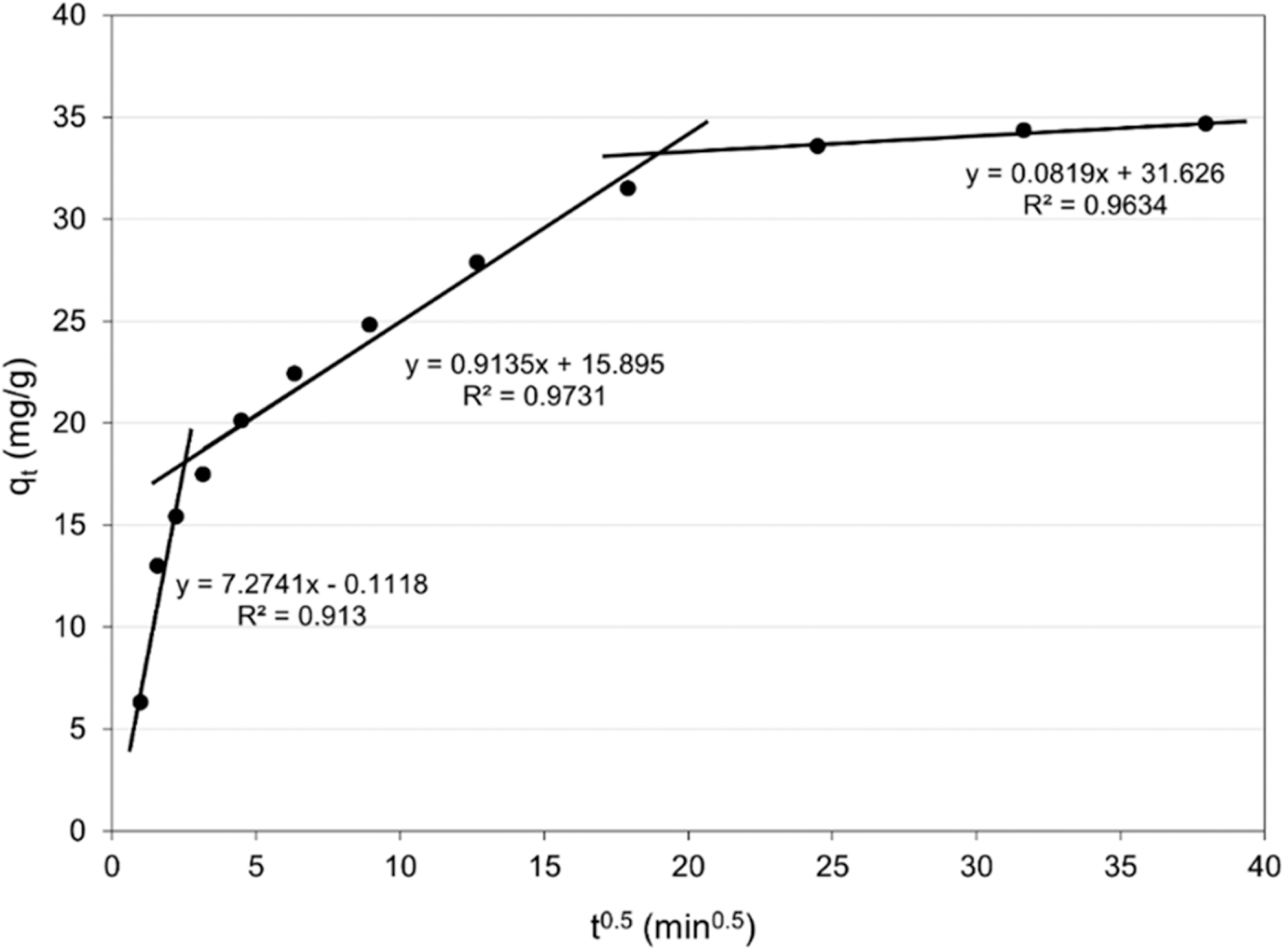
Intraparticle diffusion plots for Co(II) ions sorption on polyampholyte resin.

The Weber–Morris model, defined as an intraparticle diffusion model, was utilized to identify the stage governing the overall sorption process of Co(II) ions on the resin. The nonlinear trajectory of the graph of *q*_t_ against the square root of time highlights the multistage nature of the sorption process. Figure 11 illustrates three distinct stages in the sorption process of Co(II) ions on the resin. Initially, diffusion takes place within the boundary layer (first stage). The second stage signifies external surface sorption, while the third stage corresponds to diffusion into the interior of the grain, representing the final stage of the equilibrium state. During this stage, sorption becomes very slow and stable, reaching its maximum value.^67^ If the graph had only one limiting stage, it would pass through the origin of the coordinate system.^68^ The rate constants (*k*_D_) for the first, second and third stages are 7.274, 0.9135, and 0.0819 mg/(g min^1/2^), respectively. Analyzing the calculated rate constants for each stage reveals that the stage controlling the process rates is the third stage, with the smallest *k*_D_ value indicating diffusion into the resin grain.

#### Regeneration of Resin

After the ion exchange process and subsequent resin drying, a noticeable change in color from orange to pink was observed. Following sorption, the resin underwent treatment with various regeneration agents, including strong acids, a strong base and demineralized water. The concentration of Co(II) ions in the solution and the recovery of ions after regeneration were then assessed. The results obtained are summarized in Table 5.

**Table 5:** Results Obtained during Polyampholyte Resin Regeneration Using Various Agents

regeneration agent	concentration of Co(II) ions [mM]	recovery [%]
6 M HCl	17.80	89.00
1 M HCl	18.02	90.11
2 M H_2_SO_4_	19.63	98.17
1 M H_2_SO_4_	17.44	87.22
1 M NaOH	0.07	0.34
H_2_O	0.48	2.38

The highest recovery was achieved when using strong acids as regeneration agents, with sulfuric acid demonstrating the most efficiency. The optimal regeneration for the resin, following the sorption of Co(II) ions, was observed with a 2 M sulfuric acid solution, resulting in the recovery rate exceeding 98%. The tested resin structure contains numerous amine groups that become protonated at low pH. Protonated amine group can complex chloride ions, impeding the regeneration of Co(II). Consequently, regeneration was less effective for hydrochloric acid than sulfuric acid and a higher concentration of hydrochloric acid resulted in a lower degree of regeneration.^69^ A 1 M sodium hydroxide solution exhibited the weakest regeneration, with a recovery rate of less than 0.5%. The use of sodium hydroxide proved ineffective due to the limited involvement of amine groups in the sorption of Co(II) ions. Recovery using water was modest, approximately 2%, potentially attributed to inadequate resin rinsing after sorption.

### Mechanism of Co(II) Ions Removal on Polyampholyte Resin

**Figure 12:**
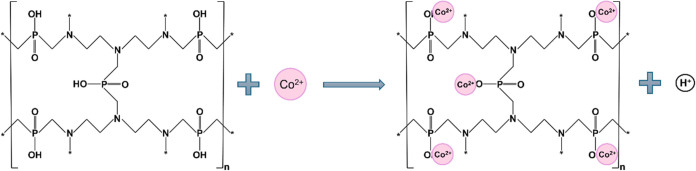
Mechanism of Co(II) ion removal on polyampholyte resin.

The mechanism of Co(II) ion removal by the polyampholyte resin, synthesized through the phosphinomethylation of diethylenetriamine, is primarily based on chemisorption, as confirmed by kinetic studies. The Weber–Morris model further suggests that diffusion is the rate-limiting step in the overall process. FTIR analysis (Figure 3) and XPS analysis (Figure 4) revealed the presence of phosphine and phosphonium groups within the resin, which actively contribute to the removal of Co(II) ions. The observed decrease in solution pH during the resin’s contact with Co(II) ion-containing solutions indicates that H^+^ ions are released by the resin in exchange for Co(II) ions, as illustrated in Figure 5B. This pH change supports the hypothesis that chemisorption is the predominant mechanism, with two plausible pathways for Co(II) binding on the polyampholyte material: (i) formation of a coordination bond between the Co(II) ion and the amine group, facilitated by the lone pair of electrons on the nitrogen atom, and/or (ii) ion exchange via P–O–H groups.^20,70^ The potential interactions between Co(II) ions and counterions on the resin can be represented as shown in Figure 12.

**Figure 13:**
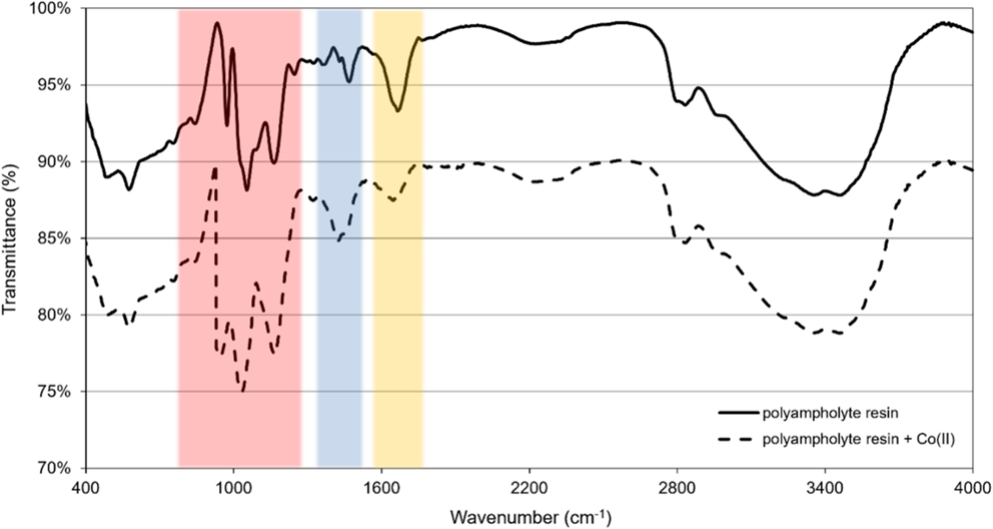
Comparison of FTIR spectra of polyampholyte resin before and after sorption of Co(II) ions.

Three distinct ranges indicate changes when comparing the FTIR spectra obtained for the polyampholyte resin and the resin after exposure to a solution containing Co(II) ions (Figure 13). The first range, highlighted in yellow, shows a lower intensity and a slight shift in the deformation band corresponding to P–OH vibrations (1659 cm^–1^). The second range, in blue, displays a higher intensity of the asymmetric vibration band of −CH_2_ and −CH_3_ groups (1426 cm^–1^). From the red shift of P–OH vibrations and the stretching bands corresponding to the vibration of P–OH (1035 cm^–1^) and P = OH (1169 cm^–1^) groups in the FTIR spectrum after sorption of Co(II) ions (Figure 13), it verified the bonding between Co ions and hypophosphite groups. When hypophosphite groups coordinate with Co ions, their electron density will decrease (due to flowing to Co ions), which leads to weakening of their bonds resulting in red shifts of IR bands.

## Conclusions

In conclusion, this study evaluated the effectiveness of a novel polyampholyte resin synthesized via the polycondensation of formaldehyde, phosphinic acid, and diethylenetriamine for purifying aqueous solutions containing Co(II) ions. The resulting polyampholyte resin exhibits chemical stability, insolubility, and high swelling properties in water. Key process parameters influence its sorption capacity toward Co(II), including pH, contact time, temperature, Co(II) ion concentration, and resin dosage. The obtained data indicate that the resin achieves optimal efficiency in removing Co(II) ions from aqueous solutions with a pH above 7.0, *T*: 328 K and after 24 h. The optimal resin dosage ranges from 4 to 8 g/dm^3^, depending on the specific application. The pseudo-second-order kinetic model fits the experimental data well (*R*^2^ = 0.999), suggesting that Co(II) ion removal occurs primarily through chemisorption, with diffusion within the resin grains as the rate-limiting step. The Langmuir isotherm accurately describes the equilibrium data, with maximum sorption capacities of 93.59, 128.7, 153.3, and 191.7 mg/g at 298, 308, 318, and 328 K, respectively. Thermodynamic studies reveal that the removal of Co(II) ions by the polyampholyte resin is a spontaneous and more favorable process at higher temperatures. The process is exothermic (Δ*H* = 23.42 kJ/mol), and the positive entropy value (Δ*S* = 0.0376 kJ/(mol K)) suggests a high degree of freedom of movement for Co(II) ions within the system. The polyampholyte resin also demonstrates ease of regeneration, achieving a maximum desorption rate of 98.2% using 2 M H_2_SO_4_. The use of this new polyampholyte resin represents a significant advancement in environmental protection (through the reduction of crude oil usage) and water treatment (via removal of toxic ions). Its high ion removal efficiency and ease of regeneration position it as a competitive alternative to currently available adsorbents on the market.

## Supporting Information Available

^31^P NMR and ^1^H NMR spectra (Figures S1 and S2) obtained by nuclear magnetic resonance spectroscopy, a figure showing the material’s swelling (Figure S3), and an overview of the XPS spectrum (Figure S4) (PDF)
