# Supplementary material for: Efficient Removal of Co(II) Ions from Aqueous Solutions Using Polyampholyte Resin: Synthesis, Properties, and Performance
Source: ACS Omega. 2025 Jan 23;10(4):3771–83. doi: 10.1021/acsomega.4c09127 (PMC11799983; doi:10.1021/acsomega.4c09127)
Supplement: Supplementary file 1 — ao4c09127_si_001.pdf [file ao4c09127_si_001.pdf]

SUPPLEMENTARY INFORMATION

Efficient removal of Co(II) ions from aqueous solutions using polyampholyte resin: synthesis, properties, and performance

Justyna Ulatowska [a]*, Łukasz Stala [a], Agnieszka Kowalska [a], Sylwia Haor [a], Izabela Polowczyk [a]

[a] *Wroclaw University of Science and Technology, Department of Process Engineering and Technology of Polymers and Carbon Materials, 27 Wybrzeże Wyspiańskiego Street, Wrocław, 50-370, Poland*

* Email: justyna.ulatowska@pwr.edu.pl

SUPPLEMENTARY INFORMATION

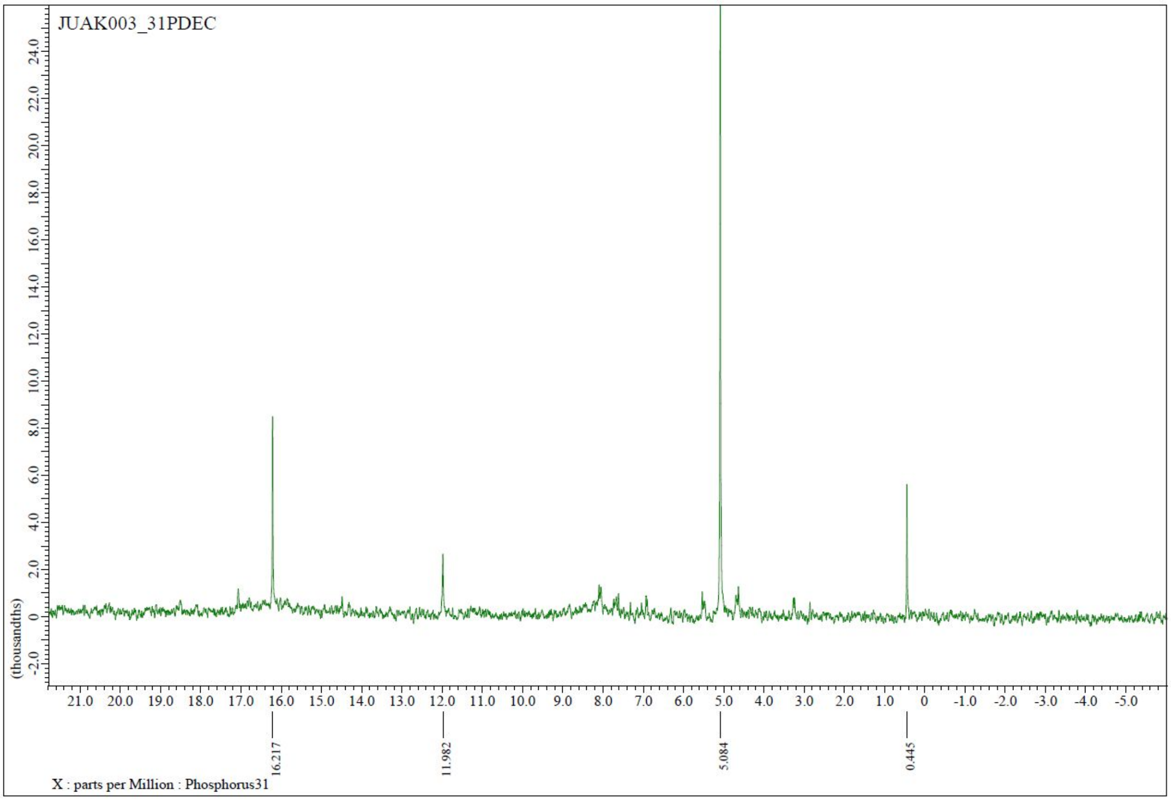

Figure S1. 31P NMR spectrum from the solution of the obtained polyampholyte resin

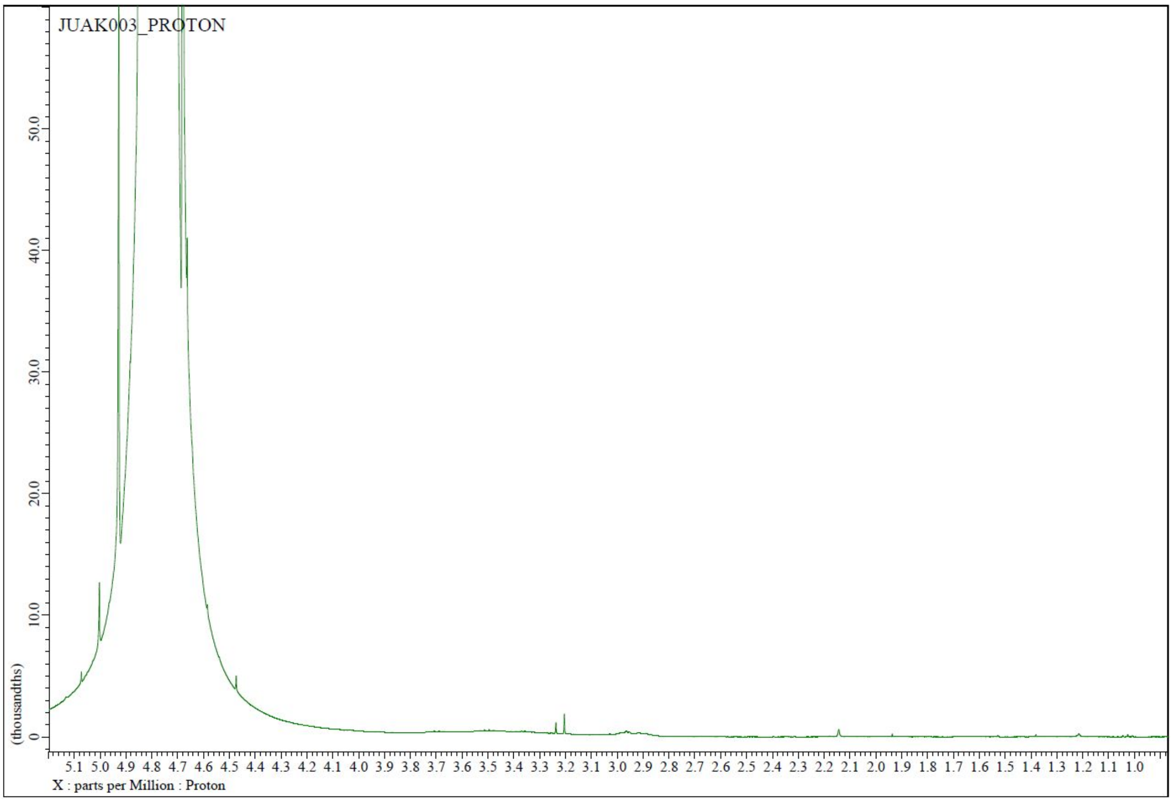

Figure S2. 1H NMR spectrum from the solution of the obtained polyampholyte resin

SUPPLEMENTARY INFORMATION

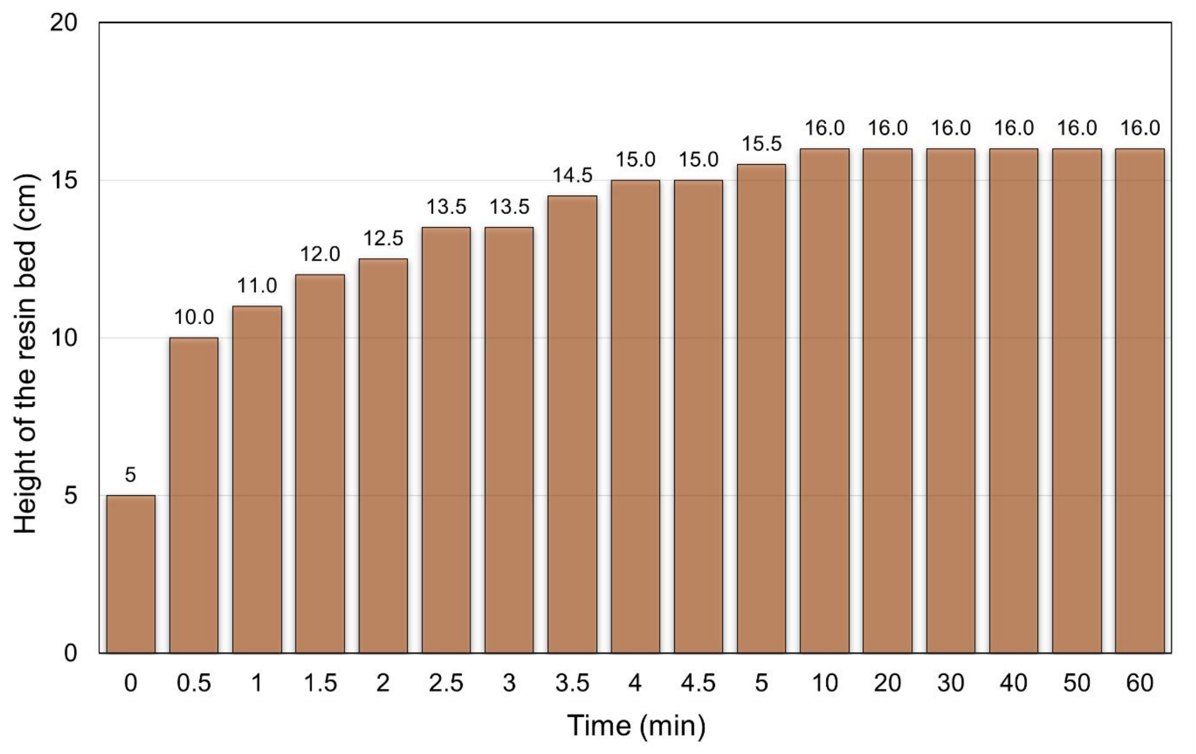

Figure S3. Change in the bed height of the polyampholyte resin during contact with a solvent.

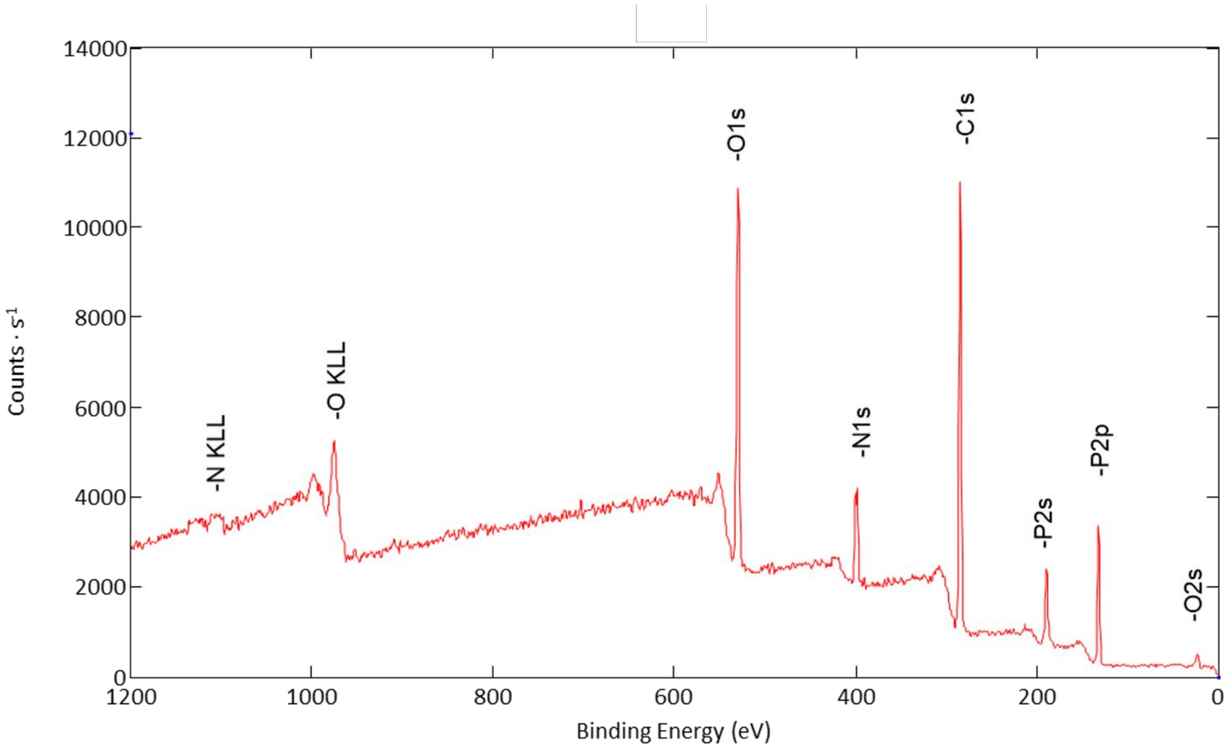

Figure S4. XPS survey profile of the polyampholyte material
